# Aspects of randomness in biological neural graph structures

**DOI:** 10.1186/1471-2202-14-S1-P284

**Published:** 2013-07-08

**Authors:** Michelle Rudolph-Lilith, Lyle E Muller

**Affiliations:** 1 Unité des Neurosciences, Information et Complexité (UNIC), Centre National de la Recherche Scientifique (CNRS), Gif-sur-Yvette, 91198, France

## 

In the past two decades, significant advances have been made in understanding the structural and functional properties of biological networks using graph-theoretic analysis. In general, most graph-theoretic studies are conducted in the presence of serious uncertainties, such as major undersampling of the experimental data. In the specific case of neural systems, however, a few moderately robust experimental reconstructions do exist, and these have long served as fundamental prototypes for studying connectivity patterns in the nervous system. Here, we provide a comparative analysis of several "historical" graphs, including areal connectivity graphs of the cat and macaque monkey cortex [1-3], as well as the neural connectivity graph of the nematode C. elegans [4,5].

While it is a common practice in applying graph-theoretic measures to empirical data to symmetrize the connectivity matrix prior to analysis, here we work with the graphs both in their unmodified directed, and symmetrized undirected forms, focusing on simple structural characterizations of their connectivity. This characterization includes the node degree distributions, the structural equivalence of graph nodes, as well as a nearest neighbor degree and assortativity analysis. All utilized measures are defined for directed graphs, but yield their forms known from the literature when applied to undirected graphs [6,7].

We find that the investigated networks share a strong component of randomness in their structural makeup, suggesting a mechanism of their formation which is much less constrained than that required for scale-free graphs. Specifically, fits of the node degree distributions are in accordance with a gamma model, supporting the idea of a simple local mechanism responsible for generating neural graphs. Secondly, the Euclidean distance of node adjacencies and node degree correlations are consistent with a independent random distribution of node connections for different nodes, but with strong correlations between in-coming and out-going connections for the same node. Finally, we find a weak disassortative tendency in the considered graphs, suggesting that in biological neural graphs nodes tend to be connected with nodes of higher average node degree.

In conclusion, our results suggest that the structural makeup of biological neural graphs has a very strong random component, in contrast to previous studies promoting the view of dominant structural features such as scale-freeness. However, the observed graph structures differ from that of the Erdos-Renyi random graphs most widely used in the computational literature, by their specific node degree distribution and strong correlations of in-coming and out-going connections for individual nodes.
